# Community genetics approaches in the prevention of beta-thalassemia: towards achieving ‘Zero beta-thalassemia’ status in India

**DOI:** 10.1186/1755-8166-7-S1-I46

**Published:** 2014-01-21

**Authors:** V R Rao

**Affiliations:** 1 Department of Anthropology, University of Delhi, Delhi, India

## 

The estimates of prevalence of β-thalassemia in India are grossly underestimated, as it is based on 0-5% carrier frequency, while population surveys indicated communities with as high frequency as 24%. The overall carrier frequency distribution of β-thalassemia, is highly heterogeneous with wide geographical foci of high risk communities with 8% and above carrier frequency all over the country. As of now, there is no mechanism to evaluate whether the frequencies are increasing or decreasing in the populations. Prenatal diagnosis and prevention of births of β-thalassemia homozygotes is the most preferred approach adopted in India. However, the extent of distribution and the occurrence across various stratification in the society with large component of rural masses, it is difficult to assume that prenatal diagnosis alone can bring in ‘Zero’ β-thalassemia status in India. Countries like Cyprus could achieve such status where large scale population carrier screening programs involving education and premarital counselling were also effectively employed besides prenatal diagnosis. In India, there is urgent need to initiate large scale population carrier screening of high risk communities along with education and awareness.

The extent of β-thalassemia distribution, identifying high risk communities and geographical regions presenting a population design for carrier screening program in India, will be presented.

